# Limitations of using feline coronavirus spike protein gene mutations to diagnose feline infectious peritonitis

**DOI:** 10.1186/s13567-017-0467-9

**Published:** 2017-10-05

**Authors:** Emily N. Barker, Angelica Stranieri, Chris R. Helps, Emily L. Porter, Andrew D. Davidson, Michael J. Day, Toby Knowles, Anja Kipar, Séverine Tasker

**Affiliations:** 1 School of Veterinary Sciences, University of Bristol, Bristol, UK; 2 University of Milan, Milan, Italy; 3 School of Cellular and Molecular Medicine, University of Bristol, Bristol, UK; 4 Institute of Veterinary Pathology, Vetsuisse Faculty, University of Zurich, Zurich, Switzerland; 5 Institute of Global Health, Faculty of Health and Life Sciences, University of Liverpool, Liverpool, UK

## Abstract

Feline infectious peritonitis (FIP) is a fatal disease of cats, and a sequela of systemic feline coronavirus (FCoV) infection. Mutations in the viral spike (S) gene have been associated with FCoVs found in tissues from cats with FIP, but not FCoVs found in faeces from healthy cats, and are implicated in monocyte/macrophage tropism and systemic spread. This study was designed to determine whether S gene mutation analysis can reliably diagnose FIP. Cats were categorised as with FIP (*n* = 57) or without FIP (*n* = 45) based on gross post-mortem and histopathological examination including immunohistochemistry for FCoV antigen. RNA was purified from available tissue, fluid and faeces. Reverse-transcriptase quantitative-PCR (RT-qPCR) was performed on all samples using FCoV-specific primers, followed by sequencing of a section of the S gene on RT-qPCR positive samples. Samples were available from a total of 102 cats. Tissue, fluid, and faecal samples from cats with FIP were more likely to be FCoV RT-qPCR-positive (90.4, 78.4 and 64.6% respectively) than those from cats without FIP (7.8, 2.1 and 20% respectively). Identification of S gene mutated FCoVs as an additional step to the detection of FCoV alone, only moderately increased specificity for tissue samples (from 92.6 to 94.6%) but specificity was unchanged for fluid samples (97.9%) for FIP diagnosis; however, sensitivity was markedly decreased for tissue (from 89.8 to 80.9%) and fluid samples (from 78.4 to 60%) for FIP diagnosis. These findings demonstrate that S gene mutation analysis in FCoVs does not substantially improve the ability to diagnose FIP as compared to detection of FCoV alone.

**Electronic supplementary material:**

The online version of this article (doi:10.1186/s13567-017-0467-9) contains supplementary material, which is available to authorized users.

## Introduction

Feline coronavirus (FCoV) infection is ubiquitous in domestic cats, with up to 90% of cats within multicat households being infected [1, 2]. The majority of FCoV infections are asymptomatic or are associated with mild intestinal disease. However, an estimated 1 to 5% of infected cats develop feline infectious peritonitis (FIP) [3, 4], characterised by the development of a variable combination of pyogranulomatous polyserositis, vasculitis and granulomatous lesions in organs, and an extremely high mortality rate [5, 6]. Avirulent or self-limiting FCoV infection was believed to be confined to the intestines, but we now know that healthy FCoV-infected cats can have systemic FCoV infection, albeit with lower FCoV viral loads than cats with FIP [7–10].

There are two different serotypes of FCoV, both of which can cause FIP. Serotype 1 FCoVs are wholly feline viruses, whereas serotype 2 FCoVs have arisen from recombination events between serotype 1 FCoVs and canine coronavirus (CCoV), involving genes encoding the C-terminal section of the replicase complex, spike protein, non-structural accessory proteins 3a–c, and part of the envelope protein [11]. There is worldwide geographical variation in the relative distribution of serotype 1 and 2, and co-infections can occur [12–17].

The “reference standard” for the definite diagnosis of FIP is the demonstration of FCoV antigen within histological specimens of the lesions consistent with FIP, usually by immunohistochemistry (IHC) [5]. As IHC requires an invasive sampling procedure, and has significant cost and time implications, alternative diagnostic techniques have been investigated, such as detection of FCoV and characterisation of FCoV genomic sequences, using pyrosequencing, Sanger sequencing or sequence specific hydrolysis probes. Recent studies have identified mutations in the gene encoding spike (S) protein of serotype 1 FCoVs that are implicated in monocyte/macrophage tropism [18]. Two amino acid substitutions, M1058L and/or S1060A corresponding to nucleotide mutations 23531A>T/C and 23537T>G respectively in the S gene, together distinguished FCoVs found in the tissues of FIP cats from those found in the faeces of healthy cats without FIP in > 95% of cases. A subsequent study, which compared detection of FCoV by RT-qPCR alone to detection of FCoV by RT-qPCR combined with sequence analysis to confirm the presence of nucleotide mutations 23531A>T/C and 23537T>G, concluded that the addition of an assay for S gene mutation analysis did not alter the specificity of the FIP diagnosis, which was already 100%, but did decrease the sensitivity from 9.4 to 6.3% for serum/plasma samples and from 72 to 64% for effusion samples [19]. However, we have shown that the nucleotide mutations 23531A>T/C and 23537T>G are likely to be markers of systemic FCoV infection rather than FIP per se, being present in 91% of the FCoV-positive tissue samples from cats with FIP and 89% of the FCoV-positive tissue samples from cats without FIP [20].

The aim of this study was to analyse a larger number of tissue, fluid and faeces samples from the University of Bristol FIP Biobank to assess the usefulness of S gene mutation analysis in the diagnosis of FIP.

## Materials and methods

**Figure 1:**
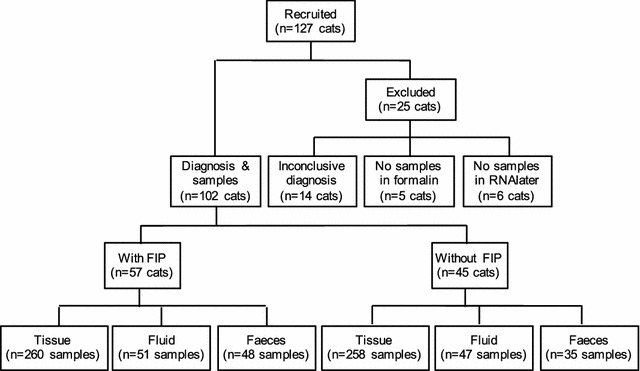
**Diagnostic pathway used.** Asterisk: Cats for which no samples were collected into formalin or RNAlater were excluded from further analysis.

See Figure 1 for the diagnostic pathway used in this study for sample selection.

### Sample collection and storage

Post-mortem tissue samples, faeces/faecal swab and body cavity fluid, if present in excess volume, were collected from cats that were euthanized with suspected FIP or due to other systemic diseases or very rarely on behavioural grounds. Some samples were included in earlier studies [20, 21]. Collection of cerebrospinal fluid (CSF), where possible, was performed immediately following euthanasia as part of a clinical training programme and was independent of presence of presenting neurological signs, which were not specifically evaluated. Where possible age and presenting signs were recorded.

Tissue samples were collected into RNAlater (Life Technologies) within 2 h of death, as per manufacturers’ instructions, and stored at −80 °C pending molecular analysis. Further samples were collected into 10% neutral-buffered formalin for histological examination and IHC. The tissues collected comprised primarily mesenteric lymph nodes, liver, kidney, spleen and omentum, while other tissues (e.g. intestine, brain, lungs, pericardium, pancreas or other lymph nodes) were included based on gross pathological findings or reported clinical signs. Body cavity fluid samples (e.g. ascitic fluid, pleural fluid, pericardial fluid, aqueous humour and CSF) were collected into plain or EDTA-anticoagulant blood tubes. Where immediate storage at −80 °C was not possible, fluid was combined with RNAlater (20% v/v) upon collection and moved to long-term storage at −80 °C within 24 h to 1 week. Faecal samples were stored immediately upon receipt at −80 °C until use.

### Histological examination and immunohistochemistry for FCoV antigen

Formalin-fixed tissue samples were subjected to standard processing for histological examination. They were trimmed and routinely embedded in paraffin wax. Sections (3–5 µm) were prepared and stained with haematoxylin–eosin, and, for the confirmation of FIP, subjected to IHC for FCoV antigen as previously described [22].

### Disease category

For a cat to be assigned to the “with FIP” group, one or more tissues needed to have histopathological changes consistent with FIP, and the presence of FCoV antigen within lesional macrophages confirmed [22]. For a cat to be assigned to the “without FIP” group, gross and histopathological changes consistent with FIP needed to be absent from all tissues collected, which had to include those associated with available ante-mortem clinical findings (e.g. eye if uveitis reported). Body cavity fluid and faecal samples were classified as “with FIP” or “without FIP” on the basis of the classification of the cat from which they originated.

For cats for which FIP was considered as a differential diagnosis based on clinical history, but for which no changes supportive of FIP were present on histological examination and for which a definite alternative diagnosis was not achieved (e.g. for definitive diagnosis additional tissues should have been examined by histology), inclusion within either the FIP or without FIP groups could not be made; these cats were excluded from further analysis. Cats for which there were no samples collected into formalin or RNAlater were also excluded from further analysis.

### RNA extraction and RT-qPCR

Total RNA was extracted from 20 mg of tissue with a NucleoSpin RNA II kit (Macherey–Nagel) using methods previously described [23]. Total RNA was extracted from 100 µL body cavity fluid or 10 mg of faeces or faecal swab using either the NucleoSpin RNA II kit or using an automated platform Chemagic 360 instrument (Perkin-Elmer) in combination with Chemagic body fluids NA kit (Perkin-Elmer) eluted in 100 µL elution buffer.

Reverse transcription was performed using a MJ Mini Gradient Thermal Cycler and ImProm II Reverse Transcriptase (Promega). Ten microlitre of total RNA were combined with ImProm II 5 × Reaction Buffer, 3 mM MgCl_2_, dNTPs (0.5 mM each), random hexamers (25 ng/μL) and ImProm II reverse transcriptase in a total volume of 20 μL. The following thermal profile was used; 20 °C for 5 min, 42 °C for 30 min, 70 °C for 15 min and 10 °C hold. The resulting 20 μL of cDNA was added to 30 μL of RNase-free water and stored at −20 °C. Randomly selected samples were checked for inhibition of the RT reaction using an RNA internal amplification control. No inhibition was detected (results not shown).

Quantitative PCR was performed using: 2 × GoTaq Master Mix (Promega), 200 nM forward and reverse primers (P009/P010), 25 nM *Taq*Man probe (P1), 2.5 mM MgCl2 and 5 μL cDNA in a total volume of 25 μL; the following thermal profile: 95 °C for 2 min and 40 cycles of 95 °C for 15 s, 55 °C for 15 s and 72 °C for 15 s; in an Agilent Mx3005P qPCR System (Agilent Technologies). The primers and probe were synthesized by Metabion (Metabion International) and were described previously [24]. Fluorescence was detected at 520 nm during the extension phase. FCoV cDNA was used as a positive control and RNase-free water as a negative control. Relative FCoV copy number per reaction was calculated for positive samples, as previously described [20].

### S gene mutation analysis by pyrosequencing

Samples that were positive by FCoV RT-qPCR then underwent conventional PCR to amplify a 153 base-pair (bp) DNA fragment encompassing amino acid positions M1058L and S1060A in the S protein gene of serotype 1 FCoV, and subsequent pyrosequencing of the amplicon. Amplification and sequencing primers (Table 1) were designed using a combination of PyroMark assay design software (Qiagen), Primer3 [25] and MFold [26], and were made by Eurofins (MWG Operon) or Metabion. Degeneracies were added to the primers, and the location of the primers optimised, based upon a sequence alignment comprised of all available serotype 1 FCoV genomes (data not shown). Briefly, PCR was performed using: 2 × GoTaq Master Mix (Promega), 200 nM forward and biotinylated reverse primers (F614/R766), 5 μL cDNA in a total volume of 25 μL; the following thermal profile: 95 °C for 2 min, 40 cycles of 95 °C for 15 s, 52 °C for 20 s and 72 °C for 20 s, before being held at 10 °C; in a MJ Mini Gradient Thermal Cycler. Samples that failed to produce definitive sequence data were pyrosequenced following repeat amplification using the same PCR protocol with 50 cycles of amplification.

**Table 1:** **Primer and probe sequences used in this study**

Name	Use	Sequence (5′–3′)
P009	qPCR forward primer	AGCAACTACTGCCACRGGAT
P010	qPCR reverse primer	GGAAGGTTCATCTCCCCAGT
*Taq*Man-P1	FCoV qPCR fluorescent probe	**FAM**-AATGGCCACACAGGGACAACGC-**BHQ1**
F614	Forward pyrosequencing primer	GCHCARTATTAYAATGGCATAATGG
R766	Biotinylated reverse pyrosequencing primer	**BIO**-AAGYCTRGCYTGYACTTGCAT
S680	M1058L pyrosequencing primer	ACAGCCTCDTTAATAGGVGGTATG
S693A	S1060A pyrosequencing primer	TAGGRGGTATGGCYWTGG
FCoV S2 F1	Forward FCoV type 2 spike gene fragment amplification primer	TCTGCTGCCATCAAAATCAC
FCoV S2 R3	Reverse FCoV type 2 spike gene fragment amplification primer	CGATGTGTAAGCAATTGTCCA

qPCR: quantitative polymerase chain reaction, FCoV: feline coronavirus, FAM: fluorescein amidite, BHQ1: black hole quencher-1, BIO: biotin.

Pyrosequencing was performed as previously described [20] using either PyroMark Q24 (Qiagen) or Pyromark Q96 (Qiagen) platforms. The dispensation order of the nucleotides was defined as; CGCTCATG for nucleotide position 23531 and CGACTGC for nucleotide position 23537.

All samples sequenced were genotyped at the nucleotide position 23531. All samples wild-type (i.e. adenine) at nucleotide position 23531, and selected mutated samples, were subjected to sequencing at nucleotide position 23537. Sequencing results were classified as non-mutated FCoV or mutated FCoV on the basis of absence or presence, respectively, of nucleotide mutations 23531A>T/C (M1058L) and 23537T>G (S1060A).

### S gene mutation analysis by Sanger sequencing

Samples that failed to produce definitive pyrosequencing data were subjected to agarose gel (1% w/v) electrophoresis, using ethidium bromide stain, with EasyLadder I (50 ng/band; Bioline, London, UK) and analysed using a GelDoc-It® Imaging System (UVP LLC, Cambridge, UK) to confirm that a single amplicon of the correct size (153 bp) had been produced using primers F614/R766 (PCR as described above). Samples that produced a 153 bp amplicon were subjected to Sanger sequencing. Sequencing was performed using non-biotinylated amplification primers in a standard protocol (DNA Sequencing and Services, http://www.dnaseq.co.uk).

### Amplification of serotype 2 FCoV cDNA

Samples that were positive by FCoV RT-qPCR, with a threshold cycle ≥ 36 (relative copy number ≤ 15), but that did not generate a 153 bp amplicon using primers F614/R766 were subjected to PCR to determine the presence of serotype 2 FCoVs. Primers were designed to amplify a ≈ 1820 bp fragment encompassing the S1 region of the S protein gene of serotype 2 FCoVs, as previously described [27]. Briefly, PCR was performed using: 2 × GoTaq Master Mix (Promega), 400 nM forward and reverse primers (FCoV S2 F1/FCoV S2 R3), 5 μL cDNA in a total volume of 25 μL; the following thermal profile: 95 °C for 2 min, 45 cycles of 95 °C for 15 s, 55 °C for 20 s and 72 °C for 2 min, followed by 72 °C for 5 min before being held at 10 °C; in an Agilent Thermal Cycler (Agilent Technologies). Positive and negative PCR controls were included in each reaction. Reaction products were separated by agarose gel electrophoresis to confirm that a single amplicon of the correct size was produced. One reaction product from each cat was subjected to a standard Sanger sequencing protocol using the amplification primers as sequencing primers.

### Statistical analysis

Data [comprising: cat identification number; age; diagnosis (with FIP vs without FIP); sample type (tissue, fluid and faeces) and specific organ/body cavity of origin where applicable; histopathology result (absence vs. presence of changes consistent with FIP); IHC result (absence vs. presence of FCoV antigen in lesions); FCoV RT-qPCR result; relative FCoV copy number; S protein mutation analysis result (non-mutated vs mutated vs serotype 2 vs. failed)] were entered into a database (Excel 2008, Microsoft; Additional file 1) and exported into IBM SPSS Statistics software (version 23.0). Continuous variables within the data sets were evaluated for normal distribution using the Kolmogorov–Smirnov test. Non-normally distributed data were described as median and range (minimum and maximum values). Population proportions were compared using Chi squared test. Data evaluating FCoV relative copy numbers in tissue and faecal samples from cats with and without FIP were analysed using a multilevel modelling approach (MLwiN v3) [28] to account for the repeated measures within cats. An alternative, simplified analysis was also employed in which non-parametric Mann–Whitney U tests were used to test for differences between cats on the individual measurements. The conclusions drawn from both analyses were in full agreement (data not shown), so the simpler Mann–Whitney U test analysis is presented here. Relative copy numbers were compared between the cats with FIP and cats without FIP for different sample types (tissue, fluid and faeces). Significance was assigned at a level of *p* < 0.05.

To determine the usefulness of a combined “FCoV RT-qPCR and sequencing result” in the diagnosis of FIP: a sample was considered to have a positive result if a FCoV RT-qPCR-positive result was followed by detection of mutated FCoV, either alone or mixed with non-mutated FCoVs; and a sample was considered to have a negative result if a FCoV RT-qPCR-negative result was obtained or a FCoV RT-qPCR-positive result was obtained followed by either detection of non-mutated FCoV or a failure to sequence the target sequence. Sensitivity was defined as the number of the samples from cats with FIP that were deemed positive based on the method under test (e.g. IHC, RT-qPCR, or combined RT-qPCR and S gene sequencing) out of the total number of samples from cats with FIP. Specificity was defined as the number of samples from cats without FIP that were deemed negative based on the method under test out of the total number of samples from cats without FIP. Accuracy was defined as the number of samples from cats with FIP that were deemed positive combined with the number of samples from cats without FIP that were deemed negative based on the method under test out of the total number of samples available.

## Results

**Figure 2:**
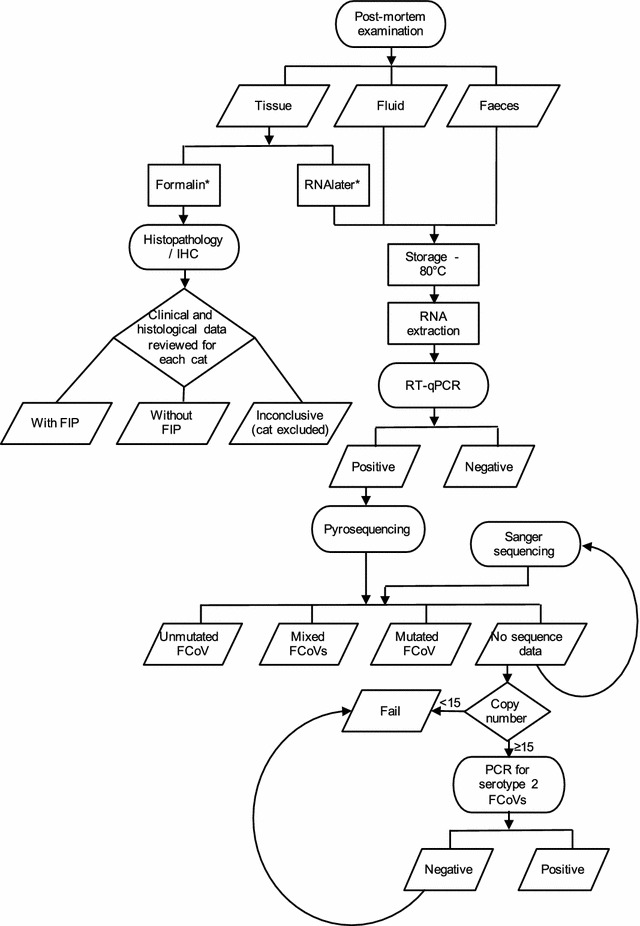
**Distribution of samples available for analysis in the Bristol FIP Biobank.**

Samples were available from 127 cats (Figure 2); for 102 of these, a definitive diagnosis was achieved (full details of the samples and results—Additional file 1): 57 cats with FIP; and 45 cats without FIP. Of the 25 cats excluded, five had no tissue samples collected into formalin, six had no tissue samples collected into RNAlater; and 14 only had limited tissue samples collected into formalin such that FIP could not be definitively diagnosed or excluded. Of the cats without FIP the reason for euthanasia and post-mortem diagnosis comprised: neoplasia in 16; inflammatory/infectious (not FIP) disease in 15; metabolic disease in four; cardiac in 1; immune-mediated in 2; miscellaneous in 6; and on behavioural grounds in 1.

Of the cats with FIP, the median age at euthanasia was 9 months (range 2–144 months; 8 cats had indeterminate age). Of the cats without FIP median age at euthanasia was 96 months (range 4–180 months; two cats had indeterminate age). Cats without FIP were significantly older than cats with FIP (U = 207, *p* ≤ 0.001). When available, 1–10 tissue samples (median 5 samples per cat) and/or 1 to 4 fluid samples (median 1 sample per cat) were examined per cat. See Table 2 for origin of samples and summary of RT-qPCR results.

**Table 2:** **Origin of samples, RT-qPCR results and threshold cycle values**

Sample source	Number of samples analysed by RT-qPCR	RT-qPCR positive result (%)	Threshold cycle for positive samples—median; range (relative copy number—median; range)
Cats with FIP			
Tissue	260	235 (90.4)	26.2; 12.4 to 39.7 (1.1 × 10^4^; 1.2 × 10^8^ to 1.2)
Fluid	51	40 (78.4)	31.2; 19.4 to 38.5 (373; 1.1 × 10^6^ to 2.7)
Body cavity fluid	35	32 (91.4)	30.9; 19.4 to 38.5 (457; 1.2 × 10^8^ to 1.2)
Abdominal	23	21 (91.3)	30.8; 22.2 to 38.5 (488; 1.6 × 10^5^ to 2.7)
Pleural	9	9 (100.0)	29.6; 19.4 to 35.0 (1.1 × 10^−3^ 1.2 × 10^8^ to 29)
Pericardial	2	1 (50.0)	38.5 (2.7)
Unrecorded	1	1 (100.0)	37.0 (7.5)
CSF	14^a^	7 (50.0)	34.8; 27.9 to 36.5 (33; 3.4 × 10^3^ to 11)
Aqueous humour	2	1 (50.0)	33.7 (6.9)
Faeces	48	31 (64.6)	30.9; 15.8 to 39.7 (457; 1.2 × 10^7^ to 1.2)
Whole faeces	42	28 (66.7)	31.0; 15.8 to 37.7 (427; 1.2 × 10^7^ to 4.7)
Faecal swab	6	3 (50.0)	30.5; 30.5 to 39.7 (598; 598 to 1.2)
Cats without FIP			
Tissue	258	20 (7.8)	36.2; 26.4 to 38.5 (12.9; 9.4 × 10^3^ to 2.7)
Fluid	47	1 (2.1)	36.4 (11)
Body cavity	28	1 (3.6)	36.4 (11)
Abdominal	13	1 (7.8)	36.4 (11)
Pleural	12	0	
Pericardial	3	0	
CSF	19	0	
Faeces	35	7 (20.0)	
Whole faeces	33	7 (20.0)	33.6; 25.7 to 38.6 (74; 1.5 × 10^4^ to 2.6)
Faecal swab	2	0	33.6; 25.7 to 38.6 (74; 1.5 × 10^4^ to 2.6)

^a^All but one sample was obtained from the cerebellomedullary cistern, one sample was collected from the lumbar subarachnoid space.

Of the 320 FCoV RT-qPCR-positive samples subjected to S gene mutation analysis 272 (85.0%) were successfully pyrosequenced (*n* = 89 at nucleotide 23531 alone, for M1058L; *n* = 183 at nucleotides 23531 and 23537, for M1058L and S1060A), 21 (6.6%) required Sanger sequencing, and 27 (8.4%) failed target gene sequencing. For the outcome of S gene analysis of FCoV RT-qPCR-positive samples see Table 3 (individual results can be found in Additional file 1).

**Table 3:** **Outcome of target gene sequencing of RT-qPCR FCoV-positive samples**

Sample source (number of RT-qPCR positive samples)	Outcome of target gene sequencing	Sequencing result (number of samples; median, and range, relative copy number per reaction)
Cats with FIP		
Tissue (*n* = 222)^a^	Success (*n* = 206)	Non-mutated FCoVs (*n* = 4; 337, 2.6 × 10^5^ to 5.4) Mixed non-mutated and mutated FCoVs (*n* = 12; 1.1 × 10^3^, 2.3 × 10^6^ to 33) M1058L (*n* = 10; 731, 4.4 × 10^5^ to 33) Double-mutated FCoVs (M1058L and S1060A) or mixed mutated FCoVs (*n* = 2; 2.3 × 10^6^ and 7.6 × 10^4^) Mutated FCoVs (*n* = 190; 1.3 × 10^4^, 1.2 × 10^8^ to 1.2) M1058L (*n* = 186; 1.4 × 10^4^, 1.2 × 10^8^ to 1.2) S1060A (*n* = 4; 1.8 × 10^4^, 9.0 × 10^6^ to 4.2 × 10^3^)
	Failure (*n* = 16)	Low copy number (*n* = 4; 6.2, 19 to 4.1) Presence of serotype 2 FCoVs (*n* = 12; 8.3 × 10^3^, 2.5 × 10^7^ to 41)
Fluid (*n* = 39)^b^	Success (*n* = 34)	Non-mutated FCoVs (*n* = 4; 190, 1.3 × 10^3^ to 41) Mixed non-mutated and mutated (M1058L) FCoVs (*n* = 4; 15, 1.1 × 10^3^ to 5.8) Mutated FCoVs (*n* = 26; 457, 1.1 × 10^6^ to 2.7) M1058L (*n* = 25; 457, 1.1 × 10^6^ to 2.7) S1060A (*n* = 1; 3.2 × 10^3^)
	Failure (*n* = 5)	Low copy number (*n* = 1; 2.7) Presence of serotype 2 FCoVs (*n* = 4; 639, 2.0 × 10^4^ to 27)
Faeces (*n* = 31)	Success (*n* = 29)	Non-mutated FCoVs (*n* = 13; 1.1 × 10^5^, 1.2 × 10^7^ to 285) Mixed non-mutated and mutated (M1058L) FCoVs (*n* = 2; 1.9 × 10^4^ and 91) Mutated (M1058L) FCoVs (*n* = 14; 63, 1.7 × 10^4^ to 1.2)
	Failure (*n* = 2)	Presence of serotype 2 FCoVs (*n* = 2; 178 and 27)
Cats without FIP		
Tissue (*n* = 20)	Success (*n* = 17)	Non-mutated FCoVs (*n* = 2; 2.1 × 10^3^ and 5.0) Mixed non-mutated and mutated (M1058L) FCoVs (*n* = 1; 2.7) Mutated (M1058L) FCoVs (*n* = 14; 63, 9.4 × 10^3^ to 4.7)
	Failure (*n* = 3)	Low copy number (*n* = 3; 5.0, 11 to 4.7)
Fluid (*n* = 1)	Success (*n* = 1)	Mutated (M1058L) FCoVs (*n* = 1; 11)
Faeces (*n* = 7)	Success (*n* = 6)	Non-mutated FCoVs (*n* = 6; 330, 1.5 × 10^4^ to 19)
	Failure (*n* = 1)	Low copy number (*n* = 1; 2.6)

^a^Sequencing was not performed on 13 FCoV RT-qPCR positive tissue samples from FIP cats as no corresponding tissue in formalin were available for analysis.

^b^One FCoV RT-qPCR positive fluid sample was lost from analysis.

### Tissue sample analysis

Tissues from cats with FIP were more likely to be FCoV RT-qPCR-positive than tissues from cats without FIP (see Table 2; 90.4% (235/260) vs. 7.7% (20/258); χ^2^ = 353.8, *p* ≤ 0.001), and with a greater relative FCoV copy number (median 8.3 × 10^3^ vs. 25; U = 2161.5, *p* ≤ 0.001).

#### Cats with FIP

Immunohistochemistry data were available for 224 of the 260 tissue samples, histopathological data alone were available for one further tissue sample and no histopathological data were available for 35 tissue samples from cats with FIP. Of the 57 cats with FIP, 56 (98.2%) had at least one tissue sample that was FCoV RT-qPCR-positive. One cat (#74) with severe pyogranulomatous lymphadenitis and pleuritis in association with FCoV antigen in intralesional macrophages, consistent with FIP, tested negative for FCoV by RT-qPCRs on all tissues. However, the use of alternative PCR primers detected FCoV in three of the four tissue samples available for analysis, whilst sequencing revealed mutations at the RT-qPCR primers (P009; P010) and probe (P1) binding sites. Four other tissue samples from three cats (#61, #94, and #96) that had histopathological changes consistent with FIP, and were positive for FCoV antigen by IHC, but negative for FCoV by RT-qPCR, had limited, focal changes as confirmed by the histological examination. Tissues with histopathological changes (incl. IHC) consistent with FIP were more likely (χ^2^ = 10.9, *p* ≤ 0.001) to be FCoV RT-qPCR-positive (132/139) than those without (69/85), and with a greater relative FCoV copy number (U = 2472.5, *p* ≤ 0.001; Table 4).

**Table 4:** **Results of FCoV antigen immunohistochemistry and FCoV RT-qPCR for tissue from cats with FIP and cats without FIP**

Tissue source	RT-qPCR	Immunohistochemistry		Total
		Negative^a^	Positive	
Cats with FIP				
	Negative	16	7	23
	Positive (relative copy number: median, range)	69 (1.2 × 10^3^, 1.4 × 10^7^ to 1.6)	132 (4.9 × 10^4^, 1.2 × 10^8^ to 1.2)	201
	Total	85	139	224
Cats without FIP				
	Negative	162	0	162
	Positive (relative copy number: median, range)	19 (16, 9.4 × 10^3^ to 2.7)	0	19
	Total	181	0	181

^a^This includes 11 tissue samples with histopathological changes consistent with FIP, from cats definitively diagnosed with FIP based on analysis of additional tissue, but for which immunohistochemistry was negative (10 were positive for FCoV by RT-qPCR).

S gene mutation analysis was performed on 222 of the 235 FCoV RT-qPCR-positive samples, of which 16 (7.1%) failed target gene sequencing. Four of the 16 samples that failed target gene sequencing were collected from two cats (#45 and #127) for which analysis of other tissue samples indicated the presence of mutated virus. These four samples had very low relative FCoV copy numbers (relative FCoV copy number ≤ 19; see Table 3). Twelve of the 16 samples that failed sequencing were from five cats (#82, #92, #145, #146, and #147), all tissues had relative FCoV copy numbers (≥ 41; see Table 3) that were expected to be successful at sequencing; additional PCRs of these samples revealed the presence of serotype 2 FCoV. Only one of these cats (#92) had evidence of a non-mutated, serotype 1 FCoV in another tissue sample. All of the cats found to have serotype 2 FCoVs were either resident in Greece (#145, #146, and #147), were imported to the UK from Greece (#82), or were suspected of having been in contact with a cat imported to the UK from Greece that was euthanased with suspected FIP (#92). The origin of the remainder of the cats contained within the Bristol FIP Biobank, where known, was the UK. Four tissue samples from three cats (#92, #97 and #103) had non-mutated FCoVs present in mesenteric lymph node (*n* = 2), liver (*n* = 1) and/or spleen (*n* = 1): one cat (#97) had mutated FCoV in another tissue sample; one cat (#103) had both mutated and non-mutated FCoVs detected in other tissues; and one cat (#92) had serotype 2 FCoV in another tissue sample. Ten tissue samples from three cats (#43, #70 and #103) had sequence data consistent with the presence of both non-mutated and mutated (nucleotide 23531A>T/C) FCoVs, these comprised mesenteric lymph node (*n* = 1), liver (*n* = 3), omentum (*n* = 2), spleen (*n* = 2), lung (*n* = 1) and kidney (*n* = 1): one cat (#103) had non-mutated FCoV sequence in another tissue sample; and one cat (#43) had mutated FCoVs detected in other tissues. Two tissue samples from one cat (#79) had sequence data consistent with the presence of either non-mutated and double-mutated (nucleotides 23531A>T and 23537T>G in the same sample) FCoVs or both types of mutated FCoVs; these comprised omentum and spleen (*n* = 2), which also had mutated FCoV detected in another tissue. These mixed FCoVs were not further characterised (i.e. as double or single mutants). Only mutated FCoVs were detected in 190 samples: 186 with the M1058L mutation (nucleotide 23531A>T *n* = 151; nucleotide 23531A>C *n* = 31; mixed, nucleotides 23531A>T and 23531A>C *n* = 4); and four with the S1060A mutation. In five of the cats (8.8%; #37, #43, #70, #100, and #101) different mutations were detected in different tissues.

#### Cats without FIP

Of the 45 cats without FIP, 12 (26.7%) had at least one tissue positive for FCoV by RT-qPCR; IHC was negative for 11 of these 12 cats, and not available for the 12th cat (#80, unstable diabetic, no histopathological findings suggestive of FIP). Histopathological and IHC data were available for 181 of the 258 tissue samples, histopathological data alone were available for 77 further tissue samples. For all cats without FIP, histopathological examination did not find changes suggestive of FIP and all were IHC-negative. Of those samples with available IHC 10.5 percent were FCoV RT-qPCR-positive (see Table 4). Of the FCoV RT-qPCR-positive samples, three (15.8%) failed S gene mutation analysis and were collected from three cats (cats #80, #91 and #141); all had very low relative FCoV copy numbers (≤ 11; see Table 3).

Two tissue samples had non-mutated FCoVs present; these comprised colon and brain from one cat (#56, pyothorax and pyogranulomatous bronchopneumonia), which also had mutated FCoV in the liver. One sample (colon) from one cat (#63, central nervous system (CNS) astrocytoma) had sequence data consistent with the presence of both non-mutated and mutated (M1058L) FCoVs; no other tissues were FCoV RT-qPCR-positive. Only mutated FCoVs were detected in 14 samples, all of which had the M1058L mutation (nucleotide 23531A>T *n* = 7; nucleotide 23531A>C *n* = 7; none mixed).

### Body cavity fluid samples

Fluid from cats with FIP was more likely (χ^2^ = 58.5, *p* ≤ 0.001) to be FCoV-positive (*n* = 40/51) compared to fluid from cats without FIP (*n* = 1/47). There were too few positive samples in the cats without FIP (*n* = 1) to compare copy numbers.

#### Cats with FIP

In one cat (#94) CSF was collected from both lumbar and cisternal sites, and was FCoV RT-qPCR-positive for the cisternal sample (relative FCoV copy number 104) and negative for the lumbar sample. Brain or spinal cord from this cat was not available for histological examination.

One FCoV RT-qPCR-positive sample (#100, pleural fluid) was lost from further analysis. Of the remaining FCoV RT-qPCR-positive samples five (12.8%) failed target gene sequencing. One of these samples (#98, pericardial fluid), collected from a cat with mutated FCoV detected in tissue samples, had a very low relative FCoV copy number (see Table 3). Four were abdominal fluids from four cats (#82, #145, #146 and #147) that had relative FCoV copy numbers (≥ 27; see Table 3) that were expected to be successful for sequencing; further analysis revealed the presence of serotype 2 FCoV. These four cats also had serotype 2 FCoV detected in tissue samples.

Four fluid samples had non-mutated FCoVs detected; these comprised abdominal fluid (*n* = 3) and pleural fluid (*n* = 1) from three cats (#55, #70 and #93); two of which had mutated FCoVs in tissue samples (#55 and #93), and one of which had both non-mutated and mixed mutated FCoVs in tissue samples (#70). Four samples had sequence data consistent with the presence of both non-mutated and mutated FCoVs; these fluids from three cats (#37, #79 and #103) were of abdominal (*n* = 1), pleural (*n* = 2), and unknown origin (*n* = 1); all cats had mutated FCoVs in tissue samples. Mutated FCoVs were detected in 26 samples; 25 with the M1058L mutation (nucleotide 23531A>T *n* = 21; nucleotide 23531A>C *n* = 4); one with the S1060A mutation.

#### Cats without FIP

Only one sample of abdominal fluid from a cat (#125) with severe interstitial pneumonia was found to be FCoV RT-qPCR-positive, at a low level (relative FCoV copy number 11.3; see Table 3); sequencing revealed mutated (nucleotide 23531A>C) FCoV. Cytological analysis of this sample was not performed. This cat also had a low relative FCoV copy number (4.7) in a sample of lung tissue, which had the same S gene mutation.

### Faecal samples

Faeces from cats with FIP was more likely (χ^2^ = 16.2, *p* ≤ 0.001) to be FCoV RT-qPCR-positive (31/48) compared to cats without FIP (7/35), but with no difference in relative copy number (U = 76, *p* = 0.221; FIP, median 457.6, range 1.2 to 1.1 × 10^7^; non-FIP, median 74.2, range 2.6 to 1.5 × 10^4^).

#### Cats with FIP

PCRs of the two samples that failed sequencing (#82 and #145) revealed the presence of serotype 2 FCoV. Both of these cats also had serotype 2 FCoV detected in tissue and fluid samples.

#### Cats without FIP

The sample for which sequencing (#115) failed had a very low relative FCoV copy number (2.6; see Table 3).

### Sensitivity, specificity and accuracy

For sensitivity, specificity and accuracy of identification of FCoV by RT-qPCR alone or RT-qPCR in combination with subsequent detection of serotype 1 mutated FCoVs in the diagnosis of FIP see Table 5. There was no significant difference between cats with FIP and cats without FIP for the proportion of mutated FCoVs detected in FCoV RT-qPCR-positive tissue and fluid samples (76.2% vs. 88.9%); χ^2^ = 2.96, *p* = 0.086).

**Table 5:** **Sensitivity, specificity and accuracy of diagnosis of FIP using molecular diagnostics**

	Basis of diagnosis	Tissue^a^	Fluid	Faeces
Sensitivity % (*n* = positive/total)	RT-qPCR alone	89.8 (*n* = 202/225)	78.4 (*n* = 40/51)	64.6 (*n* = 31/48)
	Combination testing^b^	80.9 (*n* = 182/225)	60 (*n* = 30/50)^c^	33.3 (*n* = 16/48)
Specificity % (*n* = negative/total)	RT-qPCR alone	92.6 (*n* = 239/258)	97.9 (*n* = 46/47)	80 (*n* = 28/35)
	Combination testing^b^	94.6 (*n* = 244/258)	97.9 (*n* = 46/47)	100 (*n* = 35/35)
Accuracy % (*n* = true result/total)	RT-qPCR alone	91.3 (*n* = 441/483)	87.8 (*n* = 86/98)	71.1 (*n* = 59/83)
	Combination testing^b^	88.2 (*n* = 426/483)	78.4 (*n* = 76/97)	61.4 (*n* = 41/83)

The reference-standard for diagnosis of FIP was considered identification of FCoV antigen by immunohistochemistry in at least one tissue in association with appropriate histopathological changes, and cats were considered “without FIP” where FIP was excluded as a diagnosis.

^a^As some positive samples without histopathological data were not subjected to sequencing, only those samples with histopathological data available were included in these calculations.

^b^RT-qPCR in combination with spike protein sequence characterisation FCoV.

^c^One sample lost from analysis.

## Discussion

In total, 699 tissue, fluid and faeces samples were analysed from 102 cats, 57 with FIP and 45 without FIP. This is a marked increase in the number of samples analysed as compared to most previous studies [20, 21, 29, 30], and contains similar numbers of effusion samples to two previous studies [19, 31]. Some studies have examined the use of FCoV RT-PCR alone in the diagnosis of FIP using fluid samples [29, 31]. Other studies [19, 21] compared FCoV RT-PCR to FCoV RT-PCR in combination with characterisation of S gene mutations in the diagnosis of FIP using body cavity fluids, these derived similar sensitivity (72 to 85% for FCoV RT-PCR alone and 60 to 64% for FCoV RT-PCR and S gene mutations characterisation) and specificity (100% for both FCoV RT-PCR alone and FCoV RT-PCR and S gene mutations characterisation) as obtained in this study (sensitivity 78.4% for FCoV RT-PCR alone and 60% FCoV RT-PCR and S gene mutations characterisation; specificity 97.9 and 97.9% respectively).

As expected, in cats with histological changes consistent with FIP, individual tissues without FIP lesions were more likely to be FCoV RT-qPCR-negative or have lower copy numbers than those tissues in which FIP lesions were present. Overall, however, FCoV RT-qPCR was more sensitive (89.7% vs. 62.1%; calculated from Table 4) than IHC at detecting FCoV in tissues from cats with FIP. Therefore, not surprisingly, to maximise the sensitivity of both IHC and RT-qPCR at detecting FCoV in cats with FIP, biopsies should be collected from tissues with imaging changes or gross visual changes consistent with granulomata, and from lesion sites, whenever possible. Distribution of granulomatous lesions within a tissue, and virus laden macrophages within a lesion, is not uniform, which could account for the failure to detect viral antigen in some FIP lesions, and the negative FCoV RT-qPCR result in some cats in which the combined histological and IHC examination confirmed FIP. However, application of the calculated sensitivity to suspect FIP cases has to be viewed with caution, as not all samples included in the study were selected on the basis of clinical signs and gross pathological changes. In contrast, 10% of tissue samples from cats without FIP had a positive FCoV RT-qPCR result even though there were neither histological changes consistent with FIP nor was there evidence of viral antigen expression in tissue macrophages by IHC, resulting in a specificity of 90% for FCoV RT-qPCR, as compared to 100% for the reference standard of IHC. Persistence of FCoV in both intestinal and extraintestinal tissue macrophages in the absence of disease has been reported in healthy cats experimentally infected with FCoV, albeit with authors reporting IHC to be a relatively insensitive method of FCoV detection compared with RT-qPCR [7].

Two previous studies on cerebrospinal fluid only evaluated samples collected from the cerebellomedullary cistern [29, 30]. One study evaluated the use of immunocytochemistry for the diagnosis of FIP; when all samples were combined there was a sensitivity of 81% and a specificity of 85% [30]. The other study evaluated the use of RT-qPCR for the diagnosis of FIP, where the sensitivity was 42%, but specificity was 100 percent [29]. The results of the latter study are comparable with those of the current study (sensitivity 50%; specificity 100%). However, the apparent effect of the site of cerebrospinal fluid collection on diagnostic sensitivity of FCoV infection warrants further investigation. In cats with FIP there may be concern that some clinical signs could relate to increased intracranial pressure, where lumbar sampling could provide lower risk of herniation cf. cisternal sampling; however, FIP lesions rarely go beyond the leptomeninges of the brain stem (unpublished results).

Detection of FCoV by RT-qPCR in colonic tissue of 3 cats without FIP was not surprising as enteric infection has been described in otherwise clinically healthy cats [7, 8, 32]. These 3 cats were euthanized as a result of clinical signs relating to pyothorax, brain tumour and nasal adenocarcinoma, had unremarkable histopathological examination and negative IHC of colonic tissue. In the 2 of the 3 cats where faeces were available for testing there was no detectable viral shedding. In 1 cat the FCoV was non-mutated, another cat had mutated FCoV, and in the remaining cat mixed non-mutated and mutated FCoV was detected. Of note, the cat with pyothorax also had non-mutated virus detected at a low level in its brain, and mutated virus detected at low level in its liver, suggesting that it was viraemic at the time of death and that the virus persisted not only in the colon but also in extraintestinal tissue macrophages [7]. If colonic tissue samples are excluded, all but one tissue sample from the non-FIP cats that were FCoV RT-qPCR-positive for which sequence data were available (*n* = 13) had mutated FCoV, confirming our previous findings that S gene mutations are a marker of systemic spread [20].

False-negative FCoV RT-qPCR results are possible, due to the high rate of FCoV genomic mutation, which could result in inefficient binding at the primer and/or probe sites. As only samples with FCoV RT-qPCR-positive results had pyrosequencing performed, FCoV RT-qPCR-negative results were not confirmed. However, false negative RT-qPCR results are considered to be uncommon, and were only detected in 1 of 57 cats with confirmed FIP in this study where at least one tissue from all the other cats were positive.

It is possible that the calculated relative FCoV copy numbers in this study are an overestimate of viral load. FCoV RT-qPCR assays amplify both cell-associated subgenomic mRNA, as well as cell-associated or virion-associated genomic RNA, with relative abundance determined by the positioning of primers. As viral transcription starts at the 3′ end of the FCoV genome there are more subgenomic mRNAs containing viral 3′ sequence than those containing viral 5′ sequence, hence assays directed at the 5′ end of the genome (e.g. viral replicase complex genes) are less susceptible to viral load overestimation than those directed at the 3′ end of the genome (e.g. 7a/b accessory protein genes). The RT-qPCR assay used in the present study targets the region spanning the membrane-nucleocapsid gene junction (nucleotides positions 26655–26826 of FCoV isolate FIPV 79–1146 DQ010921) [24]. Overestimation is less of an issue in the faecal samples, firstly as faeces has a high level of bacteria, and is thus rich in RNases that would degrade any RNA released from cells shed into the gut lumen, and secondly as the viral RNA is most likely to be present in its virion form, which does not contain subgenomic mRNA and is protected from RNase degradation by the viral envelope. Any cell-associated viral RNA is likely to be degraded due to cell death and the abundant RNases. This study did not determine whether any faecally shed FCoV was infective; this is an important concept as faeces containing mutated FCoV theoretically has increased potential to cause FIP following faeco–oral transmission, assuming the mutated virus can enter enterocytes and replicate. Previous studies have reported 39–85% of FCoVs detected in tissues from cats with FIP had loss of 3c gene functionality [33–35], which has been associated with a loss of ability to replicate in enterocytes and therefore infectivity via the natural route [35, 36]. Enterocyte cell cultures have been used to more accurately assess FCoV infectivity and virus copy number in a recent study [8]. However, use of enterocyte cell cultures was not possible in this study, nor was 3c gene sequencing attempted.

When calculating accuracy, the authors considered it very important to include samples for which S gene mutation analysis failed, as a more accurate reflection of the clinical situation where failure to identify the presence of mutated FCoVs may be considered lack of support of the diagnosis of FIP. We were not able to obtain pyrosequencing data in all samples; this was attributed to low relative FCoV copy number, failure of amplification primer binding, or failure of pyrosequencing primers to bind to viral cDNA as a result of sequence mismatches. Conventional sequencing of the pyrosequencing amplicon was possible in 21 cases in which pyrosequencing failed (*n* = 48 of 320 samples where pyrosequencing was attempted), significant secondary structure was predicted in these amplicons at the pyrosequencing primer binding site, which could account for the failure to pyrosequence [26]. Mixed non-mutated and mutated FCoV infections may also have been missed by the pyrosequencing assay as the amplification step biases the detected sequence towards the dominant virus type. In addition, both serotype 1 and serotype 2 FCoVs have been associated with the development of FIP; however, only mutations within the S protein fusion peptide of the more prevalent serotype 1 FCoVs have been associated with systemic infection and development of FIP [18, 20]. As the recombination events that result in the formation of serotype 2 FCoVs include the S protein gene, assays that characterise this portion of the genome are not applicable to serotype 2 FCoVs. Therefore, it was predicted that pyrosequencing would fail for those samples containing serotype 2 FCoV; however, both serotype 1 and 2 FCoVs were detected by the RT-qPCR assay used in this study, as this assay targets a section of the genome not affected by the recombination events [24]. In this study only cats that failed pyrosequencing were assessed for the presence of serotype 2 FCoVs, therefore it cannot be excluded that more cats had a dual infection with both serotypes. Spike gene mutation analysis, in combination with FCoV RT-qPCR, results in a modest increase in specificity, but this is offset by a decrease in sensitivity resulting in decreased accuracy. Independent of disease prevalence within a population, there was no significant difference in the proportion of mutated FCoVs detected in tissue and body cavity fluid samples between cats with FIP and cats without FIP that were FCoV RT-qPCR-positive (χ^2^ = 2.96, *p* = 0.086). Overall, the combination of FCoV RT-qPCR and S protein mutation analysis does not enhance the diagnosis of FIP over FCoV RT-qPCR alone, and S gene mutation analysis has significant time and cost implications.

It has been previously proposed that the M1058L or S1060A substitutions could affect the fusogenic activity and cell receptor specificity of the FCoV S protein [18, 20], permitting entry into macrophages and monocytes. It is further proposed that additional FCoV mutations, along with changes within the host’s immunological response, are required to permit the seemingly uncontrolled replication seen during the development of FIP. These hypotheses are supported by the present study: firstly, the finding that the majority of FCoV detected in tissue samples from both cats with FIP and cats without FIP have M1058L or S1060A substitutions in the FCoV S protein; and secondly, as also described in a previous study of naturally infected cats [10], the finding that FCoV RNA was detected in a far greater proportion of tissue samples from cats with FIP (90.4%) than tissue samples from cats without FIP (7.8%), and, in those samples that were RT-qPCR positive, significantly higher FCoV relative copy numbers were found in the FIP samples (median 8.3 × 10^3^ vs. 25; U = 2161.5, *p* ≤ 0.001). Other viral genomic mutations have also been associated with FIP, but were not evaluated as part of this study. For example, mutations in the S protein gene encoding amino acid differences in the furin cleavage motif [37] and the heptad HR1 region of the S2 subunit [38] have been correlated with disease. Truncations of the accessory protein 3c gene have also been associated with loss of ability to replicate within enterocytes and, as such, have only been reported in FIP-associated FCoVs [35]. However, characterisation of these viral genomic mutations was not within the aims of this study, and use of these viral genomic mutations in the diagnosis of FIP has, so far, not been suggested.

Other study limitations include lack of cytological data for fluid samples, which cannot be performed retrospectively on stored samples, and was not consistently performed at the time of collection due to lack of clinical justification in euthanased cats. However, a number of other studies have examined the use of immunocytochemistry on fluid in the diagnosis of FIP [30, 39], and reported similar sensitivities for the diagnosis of FIP to the FCoV RT-qPCR used in this study, but observed increased false positive results resulting in reduced specificities. However, in our experience, false positive results are not a relevant issue, provided sufficient cell numbers can be examined; this can be achieved when cells are concentrated in cytospins or when fluids are used to prepare formalin-fixed, paraffin embedded cell pellets that can then be treated like a tissue specimen for IHC (unpublished observations; Anja Kipar) [5]. In addition, pyrosequencing was only used to target a small region of the serotype 1 FCoV genome.

The University of Bristol FIP Biobank cats included in this study represent a convenience population, including cats for which FIP was not necessarily a differential diagnosis, and cats for which the samples available do not necessarily reflect clinical signs reported and, as such, clinically relevant samples of tissues essential for a definitive diagnosis may not be included. Differences between the cats with FIP and cats without FIP in this convenience population are clearly demonstrated when age at diagnosis is compared, which reflects the diseases for which these cats were euthanased, with neoplasia being the most common reason for euthanasia in the significantly older cats without FIP. This significant difference in the age could complicate comparison of faecal FCoV shedding between each clinical group (20% for non-FIP vs. 64.6% for FIP group), as younger cats are more likely to have been recently exposed to factors that could increase likelihood of enteric FCoV infection such as multicat households (i.e. breeding cattery or rehoming shelter). One study found FCoV shedding was most common in young cats (90% in cats aged 8–56 weeks cf. 39% for cats > 56 weeks) [1]; however, another study of faecal FCoV shedding found no difference between different age categories (34.6% < 1 year of age, 31.6% 1–5 years, and 35.3% > 5 years) [40]. Ideally the cats without FIP would have been age and clinical sign matched to the cats with FIP; however, this was not possible and is generally very difficult to achieve in an unbiased clinical patient cohort. Previous case-controlled studies of FIP have either not reported age of either FIP or control cat populations [29, 31, 41], or recruited healthy control cats [42]. One advantage of the increased age in the cats without FIP is that it does imply that these cats would be unlikely to have gone on to develop FIP had their concurrent disease not resulted in euthanasia. The absence of clinically relevant samples of tissue for some cats resulted in their exclusion from analysis, as a definitive diagnosis could not be achieved.

As in earlier studies using the same methodology [20, 21], the present data suggest that the presence of M1058L or S1060A substitutions in the FCoV S protein are indicative of systemic spread of FCoV and not a definitive marker of the development of FIP. Albeit less common than mutated FCoVs in cats with FIP, mutated FCoVs may be detected in cats without FIP, and non-mutated FCoVs may be detected in cats with FIP. Despite a slight increase in specificity of 92.6 to 94.6% for tissue samples, the addition of S gene mutation analysis to the detection of FCoV by RT-qPCR in samples from cats does not help in the differentiation of cats with and without FIP, and hence, does not aid in the clinical diagnosis of FIP.

## Additional file

**Additional file 1.** **Cat code; age (in months); diagnosis (FIP or without FIP); sample type (tissue, fluid or faeces); sample origin; histopathology result (absence or presence of changes consistent with FIP); IHC result (absence or presence of FCoV antigen in lesions); FCoV RT-qPCR threshold cycle and relative copy number; S protein mutation analysis result.** n/a = not applicable, n/d = not performed, ? = unknown.

## Abbreviations

bp: base pair
CCoV: canine coronavirus
CNS: central nervous system
CSF: cerebrospinal fluid
FCoV: feline coronavirus
FIP: feline infectious peritonitis
IHC: immunohistochemistry
RT-qPCR: reverse transcriptase quantitative polymerase chain reaction
S protein: spike protein
